# Educating science editors: is there a comprehensive strategy?

**DOI:** 10.3325/cmj.2014.55.672

**Published:** 2014-12

**Authors:** Armen Yuri Gasparyan, Marlen Yessirkepov, Sergey V. Gorin, George D. Kitas

**Affiliations:** 1 Departments of Rheumatology and Research and Development, Dudley Group NHS Foundation Trust (Teaching Trust of University of Birmingham), Russells Hall Hospital, Dudley, United Kingdom *a.gasparyan@gmail.com*; 2 Department of Biochemistry, Biology and Microbiology, South Kazakhstan State Pharmaceutical Academy, Shymkent, Kazakhstan; 3 Head of the Russian Regional Chapter of the European Association of Science Editors; Chief Editor of *International Scientific Researches*, Moscow, Russian Federation; 4 Arthritis Research UK Epidemiology Unit, University of Manchester, Manchester, United Kingdom

## Abstract

The article considers available options to educate science editors in the fast-transforming digital environment. There is no single course or resource that can cover their constantly changing and diversifying educational needs. The involvement in research, writing, and reviewing is important for gaining editing skills, but that is not all. Membership in editorial associations and access to updated scholarly information in the field are mandatory for maintaining editorial credentials. Learned associations offer access to a few widely-recognized periodicals. There are also formal training courses covering issues in science writing and ethical editing, but no high-level evidence data exist to promote any of these. Networking with like-minded specialists within the global and regional editorial associations seems a useful strategy to upgrade editorial skills and resolve problems with the quality control and digitization of scholarly periodicals.

Science editors play a central role in publishing reliable research data with potential implications for practice. To gain and maintain editorial credentials, they have to be actively engaged in research, peer review, and editing. The decades-long career in editing usually starts at academic and research institutions, where the researchers are exposed to writing and editing different types of papers, posters, dissertations, and monographs (1). Surrounded by academic mentors, peers, and librarians, young scientists learn how to search through bibliographic databases, select evidence-based sources for justification of the novelty and originality of their studies, and properly structure and enhance the quality of their reports. Undertaking research by observing scientific facts, collecting data, and analyzing statistical materials bring confidence and professional credentials for future editors. At that stage of their career, they enhance their skills in writing and research ethics, and particularly in authorship, proper referencing, and transparent reporting of methodology (2,3). Publishing research data in peer-reviewed journals boosts their confidence and introduces the world of revisions. Once they publish a good paper in a widely visible journal, chances of being invited to serve as peer reviewers increase (4). With the growing reviewer experience, researchers, and primarily those affiliated to internationally recognized universities, become candidates for editorial posts. One would agree that good editors are also good authors and reviewers, who have passion toward science and writing (5) (Figure 1).

**Figure 1:**
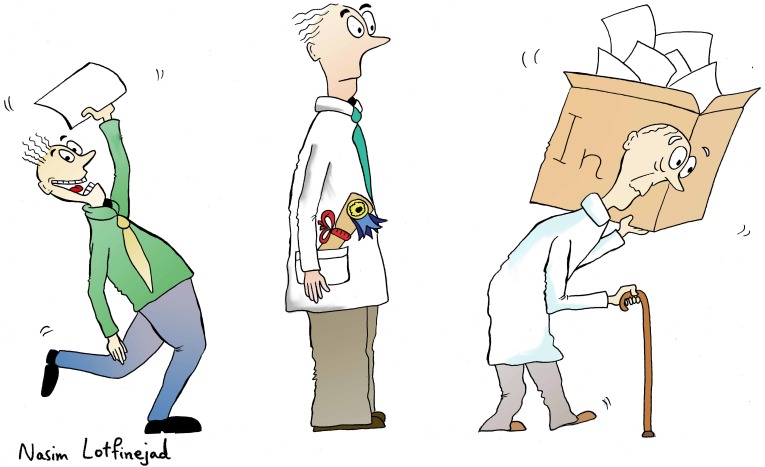
Life-long career in research and editing: from ongoing young author to credited scientist and editor overburdened with numerous incoming journal submissions

In the last decade, the publishing world has changed its landscape enormously. Functions and interests of those responsible for editing have broadened. This years’ global survey of 802 editors and publishers, organized by the International Association of Scientific, Technical & Medical Publishers, revealed that the main topic of interest in the field were open access, new models of publishing, communication, social networking, and impact metrics (6). Importantly, 90% of the surveyees did not have a recognized publishing degree, and they prioritized obtaining skills in information technologies and digital media in the coming ten years. What’s more, 53% of the surveyees emphasized the importance of reading industry news articles at least once a week, 68% – attending at least two training events per year, and more than 80% – networking through LinkedIn and Facebook.

The whole publishing industry is now oriented toward open access and archiving in digital libraries and institutional repositories, which have become the attributes of the advancement of science (7-9). More and more subscription journals switch to the online-only model, which requires advanced editorial skills for digital processing and disseminating quality information (10). The established editorial community of specialists from diverse language and professional backgrounds is gradually expanding by inviting editors with skills in digitization. Such a move is instrumental for science communication, especially in small, non-Anglophone communities, struggling to reach out to the global scientific community (11,12).

The mainstream science community can also greatly benefit from the contribution of experts in digital technologies. Properly implemented digital technologies will allow to reduce waste in research and to enhance value of the evidence base (13). As a prime example, editors and publishers who opened access to the contents of their publications in the last two decades and embarked on the technologies for detecting scientific misconduct uncovered numerous highly-cited, flawed, and otherwise unethical papers, which eventually underwent retractions (14). The so-called self-cleaning by retracting incorrect, flawed, and illegitimate papers highlighted the need for editorial skills in detecting errors at the pre-publication stage. Such skills are in high demand at both high- and low-impact journals, including the newly-launched periodicals with inadequate infrastructure and “soft” quality controls. Fortunately, researchers and editors now have an opportunity to accrue their skills in detecting and preventing unethical writings by following updates from the Retraction Watch blog (http://retractionwatch.com/) and the Jeffrey Beall’s list of predatory publishers and journals (http://scholarlyoa.com/publishers/), the two powerful educational resources that shed light on the deficiencies of the current scholarly publishing. These resources also point to the crisis in the education of all stakeholders of science communication.

A question arises as to whether formal under- or postgraduate training courses, or educational resources at all may help researchers, editors, and publishers to cope with the emerging writing and editing problems. Apparently, there is no strong evidence to promote any educational course or resource for editors. The global editorial community is diverse, and no single course or resource can cover their constantly changing and diversifying educational needs. But that does not mean that the system of education, aimed at improving editorial standards, should be abolished. On the contrary, regular topical courses for science editors with specific professional and language needs are currently strongly recommended (15). Preliminary data from the Balkans suggest that short courses of lectures on publication ethics, authorship, conflicts of interest, and scientific misconduct are helpful for creating a critical mass of researchers with ethical writing skills – best candidates for editorial posts (16,17). Modules of research ethics are especially effective when they are taught to students, who apply the gained principles in their PhD studies and related research projects (18). An experience of Croatian editors who managed to incorporate topics of writing, editing, and critically analyzing scholarly publications in the curricula of several Croatian medical schools and to organize a series of scientific communication courses also points to the positive role of educational courses (19-21). With support of the global learned associations, such as the World Association of Medical Editors (WAME), Council of Science Editors (CSE), European Association of Science Editors (EASE), and Cochrane Collaboration, Croatian editors upgraded policies of the *Croatian Medical Journal* and rebranded this small journal into a major educational resource for undergraduates, PhD candidates, researchers, and regional editors.

The above mentioned and many other regional and global editorial associations have played an important role in uniting efforts of experts and publishing didactic materials for novice and seasoned editors (22). The CSE, the oldest US-based editorial association (founded in 1957) adopted the E4 slogan – “Education, Ethics and Evidence for Editors.” The CSE published a scientific style guide and endorsed several policy statements, but perhaps the main resource of the Society, is the *Science Editor* journal. The journal stands firmly along with a few traditional periodicals in the field: *European Science Editing* (EASE), *Learned Publishing* (Society for Scholarly Publishing), and *Medical Writing* (formerly *The Write Stuff*, European Medical Writers Association). These are flagship academic journals for editors from all over the world. Members of the mentioned associations are subscribed to receive latest issues of the journals.

Over the last two years, two open-access journals covering issues in editing and publishing were launched: *Publications* (an independent journal by the Multidisciplinary Digital Publishing Institute) and *Science Editing* (Council of Asian Science Editors). These additions to the communication subject category may fill gaps in editors’ education and, as open-access sources, rapidly distribute scholarly information.

Prestigious global editorial associations, such as the CSE, EASE, and WAME, have strict selection criteria and membership fees, which can be a limiting factor for a large number of editors from developing and low-resource non-Anglophone countries. In such circumstances, regional associations can be established to develop educational resources focusing on local problems (23). One of the successful regional initiatives is the Forum of African Medical Editors (FAME), which was organized in 2003 with support of the WAME and other global associations to develop guidance for African editors (24). A strategic move of EASE to launch a sponsored membership scheme offered an alternative channel for freely distributing educational resources of the Association to editors from low-income countries. Also, several regional chapters of EASE (ie, Croatian, Italian, and Russian) were launched recently, and the first steps have been taken for expanding networks between non-Anglophone editors and organizing local educational meetings (25).

In conclusion, there are several channels for gaining editorial credentials by researchers. Those active in writing, reviewing, and publishing in most-impacting scholarly journals are likely to meet the ever-increasing criteria of skilled editors. Joining regional and global editorial associations and benefiting from their updated didactic resources is a way toward maintaining and upgrading editorial skills. Formal courses are important for basic and continuing professional development of science editors, but these have to be topical, considering the needs of specialists from certain professional, educational, and language background. One of the topics of global importance is how to run a quality open-access periodical satisfying ethical standards. No high-level evidence is currently available to advocate any specific short- or long-term course, or degree program, which necessitates more efforts for expanding the established networks of science editors and keeping them abreast of the developments in science writing and ethical editing through high-quality periodicals and regularly updated didactic materials.
